# MIS-Like Structures with Silicon-Rich Oxide Films Obtained by HFCVD: Their Response as Photodetectors

**DOI:** 10.3390/s22103904

**Published:** 2022-05-21

**Authors:** Gabriel Omar Mendoza Conde, José Alberto Luna López, Zaira Jocelyn Hernández Simón, José Álvaro David Hernández de la Luz, Godofredo García Salgado, Erick Gastellou Hernández, Haydee Patricia Martínez Hernández, Javier Flores Méndez

**Affiliations:** 1 Centro de Investigaciones en Dispositivos Semiconductores (CIDS-ICUAP), Benemérita Universidad Autónoma de Puebla (BUAP), Col. San Manuel, Cd. Universitaria, Av. San Claudio y 14 Sur, Edificios IC5 y IC6, Puebla 72570, Mexico; gaomec13@gmail.com (G.O.M.C.); imezaira@gmail.com (Z.J.H.S.); joalvada1@hotmail.com (J.Á.D.H.d.l.L.); godgarcia@yahoo.com (G.G.S.); 2 División de Sistemas Automotrices, Universidad Tecnológica de Puebla (UTP), Puebla 72300, Mexico; erick_gastellou@utpuebla.edu.mx; 3 Departamento de Ingeniería Eléctrica y Electrónica, Instituto Tecnológico de Apizaco (ITA), Fco I Madero s/n, Barrio de San José, Apizaco 90300, Mexico; haydee.mh@apizaco.tecnm.mx; 4 Facultad de Ciencias de la Electrónica (FCE), Benemérita Universidad Autónoma de Puebla (BUAP), Col. San Manuel, Cd. Universitaria, Av. San Claudio y 18 Sur, Edificio FCE1, Puebla 72570, Mexico; javier.floresme@correo.buap.mx

**Keywords:** SRO films, HFCVD, photodetector, responsivity

## Abstract

MIS-type structures composed of silicon-rich oxide (SRO), thin films deposited by hot filament chemical vapor deposition (HFCVD), show interesting I-V and I-t properties under white light illumination and a response as photodetectors. From electrical measurements, it was found that at a reverse bias of −4 V, the illumination current increased by up to three orders of magnitude relative to the dark current, which was about 82 nA, while the photogenerated current reached a value of 25 μA. The reported MIS structure with SRO as the dielectric layer exhibited a hopping conduction mechanism, and an ohmic conduction mechanism was found with low voltage. I-t measurements confirmed the increased photogenerated current. Furthermore, the MIS structure, characterized by current-wavelength (I-λ) measurements, exhibited a maximum responsivity value at 254 mA/W, specific detectivity (D*) at 2.21 × 10^11^ cm Hz^1/2^ W^−1^, and a noise equivalent power (NEP) of 49 pW at a wavelength of 535 nm. The structure exhibited good switching behavior, with rise and fall times between 120 and 150 ms, respectively. These rise and decay times explain the generation and recombination of charge carriers and the trapping and release of traps, respectively. These results make MIS-type structures useful as photodetectors in the 420 to 590 nm range.

## 1. Introduction

Silicon-rich oxide (SRO) is a multiphase material with interesting structural, electrical, and optical properties that can be tuned by silicon excess and defects in thin films [1,2,3]. SiO_x_ materials are strongly influenced by oxygen content, as it determines their optical and electrical properties, such as absorption coefficient, band gap energy, luminescence, refractive index, and electrical conductivity. In these SiO_x_ films, the oxygen content, quantified by the x-parameter, was determined taking into account the Si-O-Si asymmetric stretching vibrational modes that exist around 1050 cm^−1^ and 1150 cm^−1^. As a grown SiO_x_ material, it contained characteristic Si-O and Si-H bonds exhibited by absorption spectra *α*(*ω*). The density of H-bonds in the SiOx microstructure can be determined by using the Si-H wagging vibrational mode centered at 640 cm^−1^ [4].

By applying an annealing process, it is possible to both generate the hydrogen desorption from the films and to modify their atomic structure, which leads to changes in their optical and electrical properties. This fact has motivated extensive research by studying its fundamental optical and electronic properties. Thus, it makes this material very attractive for the manufacture of optoelectronic devices, such as photodetectors, solar cells, and light emitters [5,6,7]. The SRO films can be obtained by various techniques, such as sputtering [8,9], plasma-enhanced chemical vapor deposition (PECVD) [10,11], low-pressure chemical vapor deposition (LPCVD) [12,13], and hot filament chemical vapor deposition (HFCVD) [14,15].

On the other hand, metal-insulator-semiconductor structures with SRO as the insulator and active layer provide good results under illumination conditions [16,17,18,19]. In previous reports, it was found that the SRO-MIS structure exhibits an active optical response in the ultraviolet range due to the radiation emission of silicon nanocrystals (Si-ncs) formed in SRO films and the defects present in the mixed-phase nanostructure material [16,17,18,19]. Due to its high profitability and low cost of mass production, this material can be integrated with standard manufacturing processes in the microelectronics industry based on silicon technology.

This research analyzed the electrical and optical behavior of the SRO thin films obtained by the HFCVD technique; this one has advantages such as the easy deposition of thin films, which present good quality in terms of optical and structural properties, with a short deposition time of 3 min. Furthermore, novel photoconductive and photodetection properties were reported in the study of the Au/SRO/Si-n MIS structure. Current-voltage (I-V) curves obtained from these structures show that the maximum photocurrent occurs at a voltage of −4 V, as confirmed by current-time (I-t) measurements. Photodetector quality metrics, such as specific detectivity (D*) and noise equivalent power (NEP), were also obtained, as well as switching characteristics and transient photodetection response, where rise and decay time constants (t-rise and t-decay, respectively) were obtained from the MIS structure. The outstanding result of this analysis was the presence of excellent photodetection in the range of 420–590 nm, which leads to the fact that the MIS structure can be used as a photodetector.

## 2. Materials and Methods

Silicon-rich oxide films were deposited on 1–5 Ω-cm (1 0 0) n-type silicon substrates. Six SRO films were obtained using the HFCVD deposition system under the following parameters: a deposition time of 3 min, voltage applied to the filament at 74 V, system pressure at 1 atm, and a 6 mm distance from the filament source (dfs). Three values were used for source-substrate distance (SSD) and 50 sccm hydrogen flux levels. The SRO films were broken into two halves, one of which was thermally annealed at 1050 °C. Thermal annealing was performed in a tube furnace for 60 min in a nitrogen atmosphere. Table 1 lists the 6 SRO film labels used to identify the samples.

To obtain the optical properties of the SRO films, transmittance measurements were performed using a UV-Vis-NIR Cary 5000 spectrophotometer (Agilent Technologies, Inc., Santa Clara, CA, USA) with a wavelength range of 200–900 nm and a resolution of 0.5 nm. Photoluminescence (PL) spectra of the SRO films were obtained using a FluoroMax 3 spectrofluorometer (Horiba. Ltd., Kyoto, Japan) with a 150 W xenon excitation lamp and a high sensitivity emission detector using the following parameters: 1 nm resolution, 370 to 1000 nm range, and the excitation wavelength used was 335 nm. The thickness of the SRO films was obtained with a Dektak 150 Profilometer (Veeco, Plainview, NY, USA).

The composition of the MIS structures was as follows: For the front and rear contacts, gold films were deposited by a cathodic erosion system. The front contacts had a rectangular geometry (4 × 3 mm) and a thickness of 90 nm, and these structures were subsequently sintered at 450 °C in a nitrogen atmosphere.

**Figure 1:**
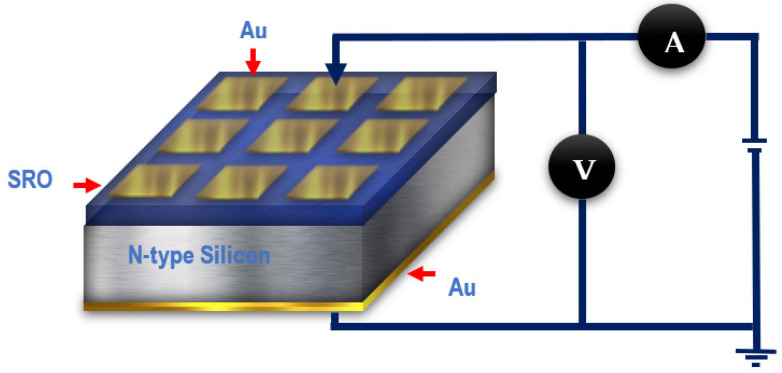
Device and circuit schematics for measuring I-V characteristics.

The I-V curves were measured with a Keithley 4200-SCS parameter analyzer system (Keithley, Cleveland, OH, USA) at room temperature, occupying a voltage step of 0.0125 V. The I-V curves were measured under different lighting conditions: dark, white light, short UV (254 nm), and long-wave UV (365 nm). The I-V characterization was performed in a vertical measurement configuration, as shown in Figure 1.

**Figure 2:**
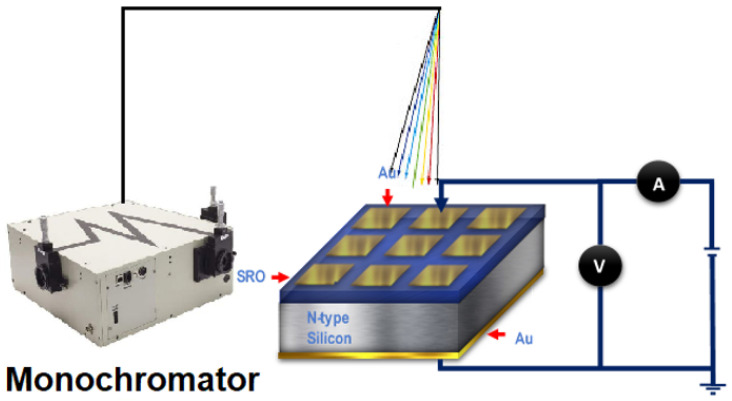
Schematic diagram for measuring I-λ characteristics.

The wavelength vs. current (λ-I) characterization of the MIS structures was performed with a Cornerstone Oriel CS260 UV-Vis monochromator (Newport Corporation, Irvine, CA, USA) and a Keithley 4200 SCS system, as shown in Figure 2. The λ-I curves under wavelength range (200–1000 nm), switching characteristics, photosensitivity, responsivity, D*, and the NEP of the Au/SRO/Si structures were measured and compared.

## 3. Results

The thickness of the SRO films was obtained by profilometry and reported in Table 2.

**Figure 3:**
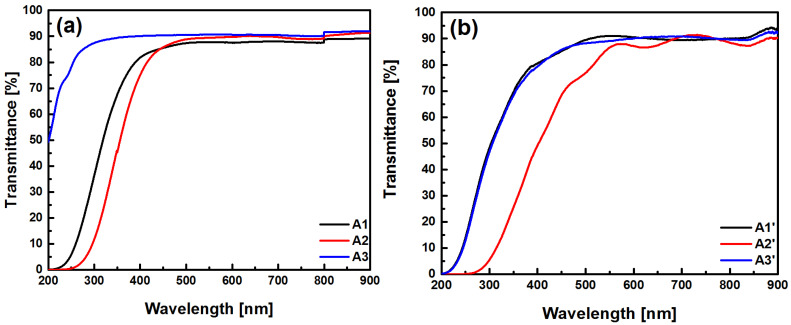
Transmittance the SRO films without (**a**) and with thermal annealing (**b**).

Figure 3 shows the UV-Vis transmission spectra of the SRO films deposited on quartz. As shown, these spectra exhibit relatively high transmittance (>80%) in the range between 400 nm and 900 nm, which drops to zero for wavelengths less than 250 nm, except for sample A3, which had a high transmittance (>85%) from 250 nm.

**Figure 4:**
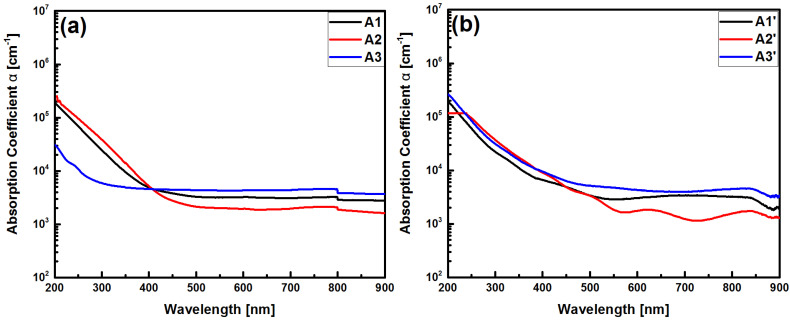
Absorption coefficients of SRO films without (**a**) and with thermal annealing (**b**).

To analyze the UV-Vis transmission spectra of the SRO films with and without annealing, the absorption coefficient and band gap energy (*E_g_*) were determined, and for this calculation, Beer–Lambert’s law was used [20]. To obtain an estimate of the band gap energy, the absorption coefficient first needs to be calculated using the following formula obtained by Beer–Lambert’s law:

α(hv)=(−ln[T(hv)])/d

where *d* is the thickness, and *T*(*hv*) is the transmittance of the film in percent. The absorption coefficients of the six SRO films are shown in Figure 4.

**Figure 5:**
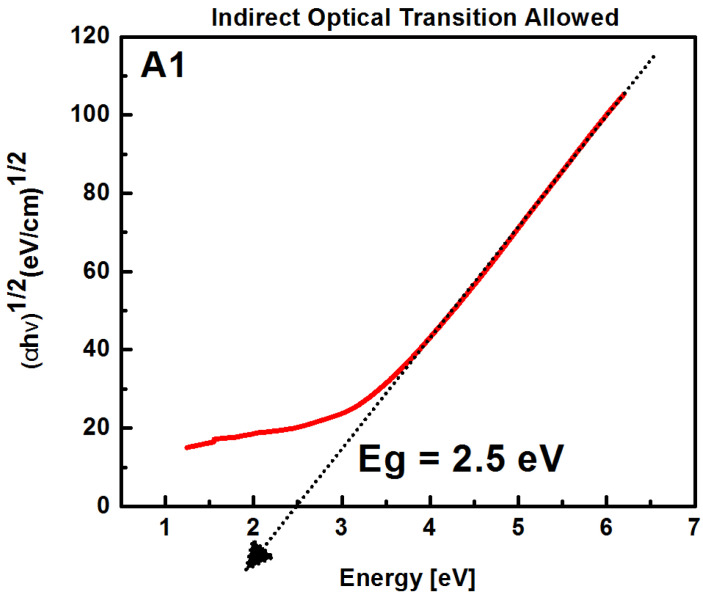
Square root of (*αhv*)^1/2^ vs. photon energy for SRO films.

The optical band gap is obtained by Tauc’s law [21], assuming (αhν)12=A(hν−Eg)*,* where *α* is the absorption coefficient, (*hv*) is the energy of the photon, *E_g_* is the forbidden energy, and *A* is a constant, using a power *n* of a value of *½*, which is the coefficient reported by SRO [1]. The *½* exponent indicates that the allowable indirect optical transitions occur mainly in mixed-phase materials. Figure 5 shows the procedure used to calculate the energy band gap (*E_g_*).

The obtained band gap energies are shown in Table 3.

For the SRO films with thermal annealing, the band gap energy increased slightly as a consequence of a restructuration of the Si-ncs that reduced their dimensions and increased their band gap due to dimensional quantum confinement [14].

**Figure 6:**
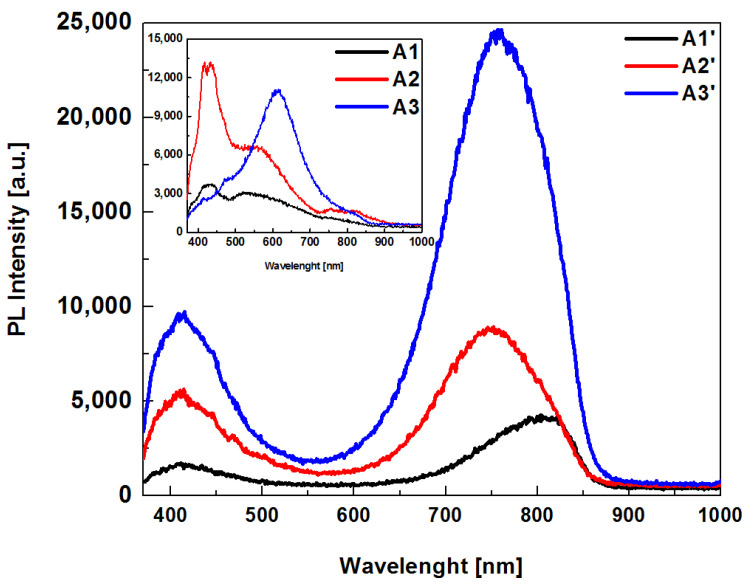
PL spectra measured from deposited SRO films. Inset shows PL spectra of unannealed SRO films.

The PL spectra are shown in Figure 6, where the inset in the figure represents the spectra obtained from the unannealed film, while the main figure shows the PL spectra obtained from the annealed one. The PL spectra after heat treatment had an emission band in the range of 650 nm to 850 nm, and the emission peak was centered at 750 nm. The shape of the emission peak of all samples was like Gaussian; the spectrum shows the intensity emission increased, exhibiting the maximum intensity for sample A3’. The red-shifted peak for the A1’ and A3 films was due to the increase of the non-bonding oxygen hole center (NBOHC ≈ 630 nm) and to the quantum confinement (QC ≈ 760 nm). The annealing also plays an interesting role, in addition to the interaction mechanism between the Si-ncs interface and the oxide matrix, which plays an important role in the emission, as well [12]. Other weaker peaks are also shown at 425 and 450 nm, which are the emission components of the PL spectra and have different origins [22,23,24].

**Figure 7:**
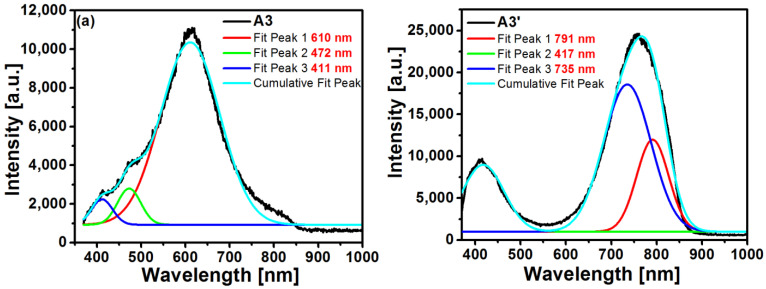
Deconvolution of PL spectra of A3 films without (**a**) and thermally annealed (**b**).

For SRO films, the two most accepted mechanisms to explain the red and infrared light emission are as follows: the first mechanism is attributed to quantum confinement (QC) effects generated in Si-ncs formed in the dioxide matrix, and the second one is due to radiative emission generated by electronic transitions between electronic states that originated due to the presence of defects formed in the atomic structure of the SRO material. For the case of the radiative emission process, two models were proposed. In the first model, the luminescence of Si-ncs may be due to the interband radiative recombination process of electron-hole pairs confined within the crystal. In the second model, the photoluminescence of SRO films is attributed to the presence of oxygen vacancy-related defects in the SiO_2_ matrix or defects in the SiO_2_/Si-nc interface [25], and this emission process is associated with certain types of defects related to the growth process, such as weak oxygen bonds (WOB), neutral oxygen vacancies (NOV), unbound oxygen vacancies (NBOHC), positively charged oxygen vacancies ($E_{\delta }^{\prime }$), *E’* centers, and oxygen vacancies located in the Si-nc/luminescent center at the SiO_2_ interface (CLI) [14,26,27]. These emission mechanisms give the PL spectra a broad form, as shown in Figure 6. To find all possible contributions to the photoluminescence process in SRO films, the Origin software was used, and as a function to carry out the deconvolution of the bands, the Gaussian function was used. The deconvolution of each spectrum was performed as shown in Figure 7 for the A3 and A3’ films.

Table 4 shows the positions of the emission peaks obtained from the deconvolution of the PL spectra and the source of emission according to their positions.

From Table 4, it can be seen that all the unannealed films showed PL emission attributed to WOB, but only the A1 and A2 films exhibited PL ascribed to $E_{\delta }^{\prime }$; similarly, only the A1 and A3 films exhibited PL attributed to the presence of NBOHC, while the A3 film with the highest SSD showed PL attributed to NOV emission. In regard to the annealed films, WOB defects and QC mainly determined the photoluminescence due to the presence of Si-ncs, although the A1’ and A2’ films exhibited additional PL due to CLI and $E_{\delta }^{\prime }$ mechanisms. The greater contribution to PL is attributed to Si-nc effects, which brings confinement effects due to a nanocrystal formation producing an increase of the band gap, which is controlled by the Si nanostructure size, in addition to the increment in PL and the red-shift of the peaks. The red-shifted peaks for the A3 and A3’ SRO films was due to NBOHC (630 nm), and also to the quantum confinement (760 nm), which, in addition to the interaction mechanism between the Si-ncs interface and the oxide matrix, also plays an important role in the emission [12].

**Figure 8:**
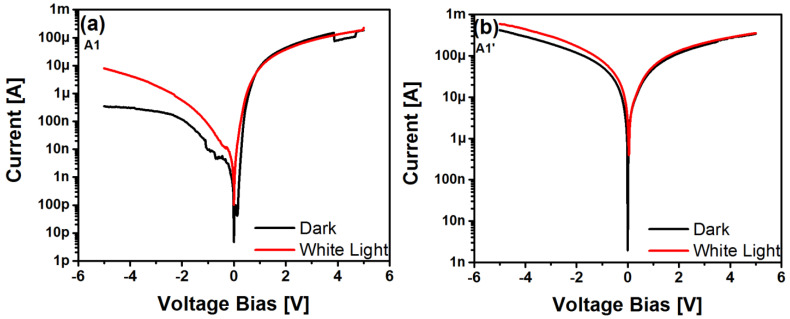
I-V curves of MIS-like structures with SRO films without (**a**) and with (**b**) annealing at the dark and white light illumination states.

On the other hand, Figure 8 illustrates the I-V curves of the MIS-like structures both under dark and white light illumination conditions. The I-V curves were generated in the structures by voltage bias in the range of 0 to 5 V and 0 to −5 V.

It was found that the electrical behavior shown in Figure 8a was similar for all structures with and without annealing. A remarkable characteristic of these structures is that they have a high on-state in the polarization from 0 V to 5 V, as well as from 0 V to −5 V. As can be seen from the I-V curves in general, they showed an increase in current when illuminated by white light; however, this increase was more pronounced for the case of the unannealed A1 film, as shown in Figure 8a, in the range of reverse bias. This outstanding characteristic is ascribed to the photodetection effect, which is described in more detail below. Furthermore, in Figure 8b, high on-states are shown in both polarizations, and there was no increase in photocurrent; this behavior was similar in all devices in which their SRO layers were annealed.

**Figure 9:**
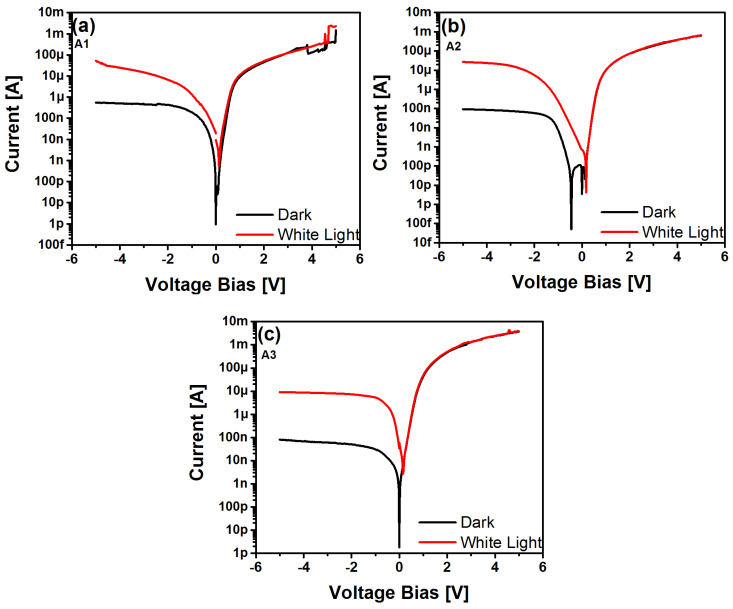
I-V curves of Au/SRO/Si structure with SRO A1 (**a**), A2 (**b**) and A3 (**c**) films under dark and white light illumination.

Figure 9a–c show the I-V curves of the MIS-like structures with SRO thin film without annealing with different SSDs (6, 7, and 8 mm, respectively). As with the I-V curves of Figure 8, in forward polarization, these structures exhibited a high conduction state under forward bias without significant increase in response when illuminated. Contrarily, under reverse bias, it was found that the response was increased up to three orders of magnitude when the structure was illuminated with respect to the dark current, which was about 10^−7^ A. The latter also indicated a small leakage current in these structures. We point out that the substantial current increment under reverse bias indicates that these MOS-like structures exhibit a photoresponse when exposed to light; this fact suggests that this type of structure has good optical sensitivity.

**Figure 10:**
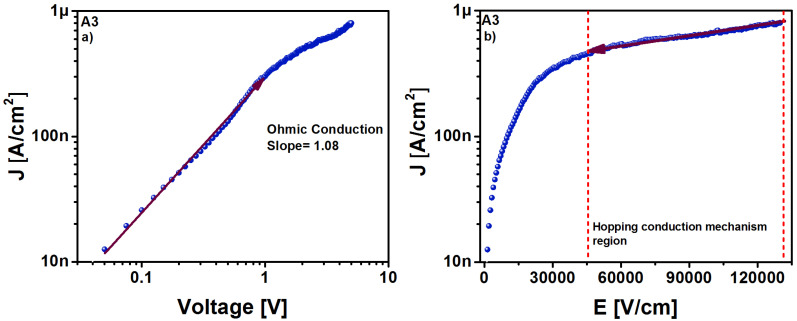
The current density in which (**a**) ohmic and (**b**) hopping conduction were present for the Au/SRO/Si structure having the unannealed SRO A3 film.

As SRO material is multiphase, the charge of the current flow in the MIS-like structure may be generated by different components. The intrinsic component of the current flow gives rise to ohmic conduction with high resistivity; for the case of when the charge carriers are injected from the gate or substrate into the SRO film, the current flow component is determined by charge carriers, which can travel freely within the oxide layer. In this case, the transport mechanism is identified as the Schottky effect (thermionic conduction), and when the charge carriers are present in the material through direct tunneling and Fowler–Nordheim tunneling or through traps, the component of the current flow is ascribed to the Poole–Frenkel effect, hopping conduction, and space charge limited current. By the analysis of the current through the structure as a function of voltage (V) and electric field (E), it was possible to identify the conduction mechanisms, which were identified to be the hopping and ohmic conduction mechanisms, as can be seen in Figure 10.

**Figure 11:**
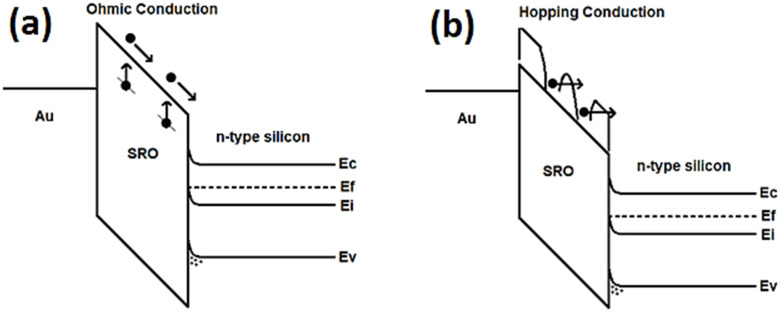
Energy band diagram of ohmic (**a**) and hopping conduction (**b**) in Au/SRO/Si structures.

The injection current corresponding to the hopping conduction mechanism is governed by Equation (1) [28]:

(1) $$J=\left(qan\nu \right)exp\left[\frac{qaE}{kT}-\frac{E_{a}}{kT}\right],\;\;\;\;\;\;$$

where *T* is the absolute temperature, *q* is the magnitude of the electron charge, *E* is the magnitude of the electric field applied through the SRO film, *k* is the Boltzmann constant, *a* is the average space in between traps, *n* is the electron concentration in the conduction band, *ν* is the vibrational frequency of the electrons in the trap site, and $E_{a}$ is the activation energy. The behavior of the current density determined by Equation (1) is located for electric fields greater than $4.5\times 10^{4}\frac{V}{\mathrm{cm}}$, which is identified as a straight line, as shown in Figure 10b. Figure 11 shows the schematic energy band diagram of ohmic, shown in Figure 11a, and hopping conduction, shown in Figure 11b. As we can see in Figure 11b, hopping conduction is due to the tunneling effect of trapped electrons hopping from one defects site to another in SRO films.

**Figure 12:**
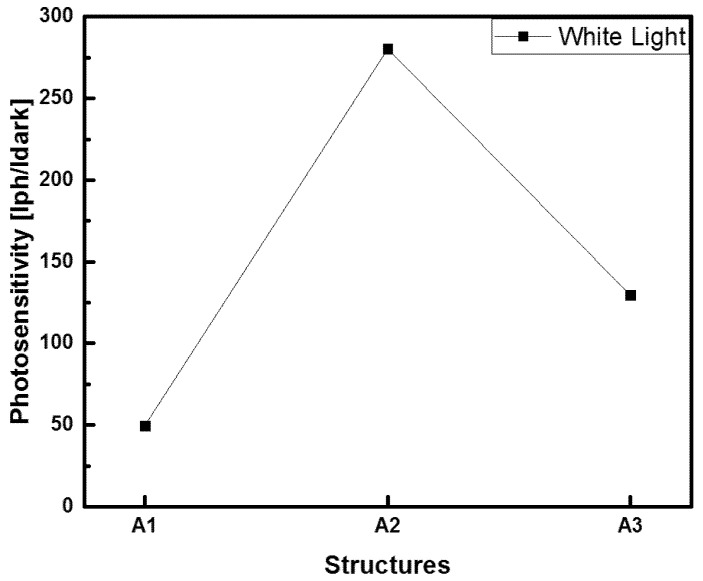
Photosensitivity of Au/SRO/Si structures at −4 V for the case of the unannealed A1, A2, and A3 SRO films.

Additionally, we explored important properties of the Au/SRO/Si structures; one such property was photosensitivity, for which the I-V curves shown in Figure 9 were used. The photosensitivity is obtained by the ratio of the photogenerated current to the dark current, which gives the device’s response to light [29]. By using Equation (2), it is possible to calculate the photosensitivity (*P*) for an operating voltage of −4 V.

(2) $$P=\frac{I_{ph}}{I_{dark}},\;\;\;$$

where *I_ph_* is the photogenerated current, and *I_dark_* is the dark one. Figure 12 exhibits three photosensitivity values obtained from of the Au/SRO/Si structures, taking into account the unannealed A1, A2, and A3 SRO films in such structures.

**Figure 13:**
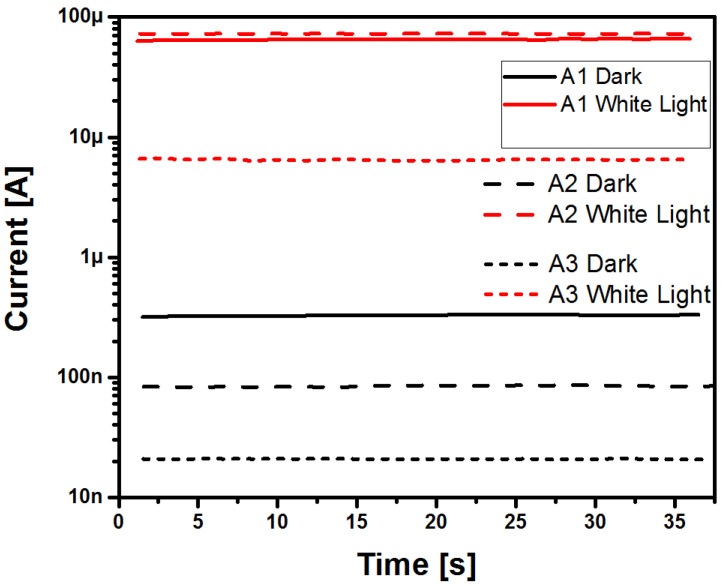
I-t curves of Au/SRO/Si structures with A1, A2, and A3 SRO films under dark and white light illumination.

The three structures had a high sensitivity to white light, reaching 50, 280, and 130 adimensional units, respectively, where the structure with the SRO A2 film possessed the highest sensitivity to white light irradiation. In order to verify the photocurrent increase shown in Figure 9, we proceeded to make I-t measurements. They were obtained under the same conditions as those of the I-V curves. These measurements were made by applying a bias voltage of −4 volts for 37 s of white light irradiation. The resulting I-t curves are shown in Figure 13.

**Figure 14:**
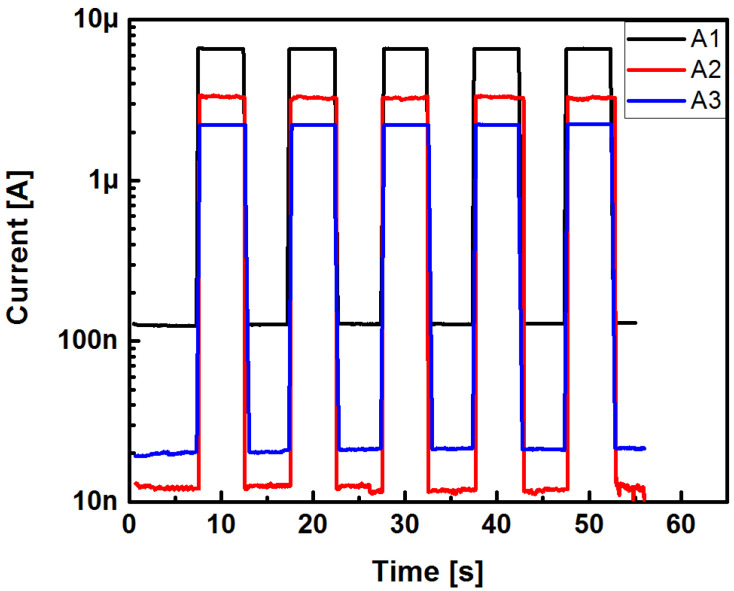
Switching response of Au/SRO/Si structures under white light illumination.

The I-t curves illustrated in Figure 13 clearly emphasize a photoresponse. When the structures were white light irradiated, the intensity of the photocurrent increments corresponded to those obtained in the I-V curves shown in Figure 9. All three structures kept a constant dark current in the order of about 10^−7^ A. It was observed from the I-t curves that when the structures were white light irradiated, a remarkable increment in the photocurrent was generated, finding that a large increment occurred of about two orders of magnitude for the case of the structures with the SRO A1 and A3 films. We point out that the Au/SRO/Si structure with the SRO A2 film showed the largest increase in photocurrent when illuminated; this increase was approximately of three orders of magnitude. The latter was corroborated with the photosensitivity values depicted in Figure 12. Figure 14 shows the switching characteristics of the three Au/SRO/Si structures, considering a reverse bias state at −4 V and under white light irradiation with an exposure time of 6 s. According to the results obtained, all structures showed very good switching performance under white light irradiation, as shown in Figure 14.

**Figure 15:**
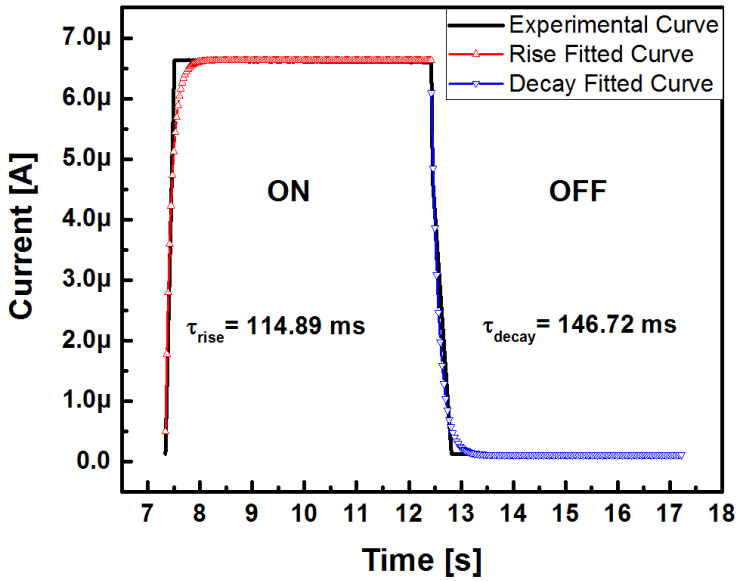
Fitted curves of the photoresponse to white light irradiation and switching response time-resolved for the Au/SRO/Si structures.

As shown in Figure 14, the curves for the three structures exhibited the same patterns, which differed only quantitatively. These currents increased by about two orders of magnitude, the dark current pulse stabilized, and equally, the photogenerated current pulse remained stable. Furthermore, the photogenerated current was observed to be as small as 2 orders of magnitude for A1, 2 1/2 orders of magnitude larger for A2, and approximately 2 orders of magnitude for A3, whilst A2 showed the largest increase in white light photoresponse. By using the current pulses from Figure 13, the parameters of rise and decay fall times (*t_rise_* and *t_decay_*) were obtained, which measured the carrier responses to the detection of the incident radiation energy into the Au/SRO/Si structures. The rise time (*t_rise_*) of a photodetector is defined as the time interval required for the current to rise from 10% to 90% of its final value in the face of a sudden change in the incident optical power in the form of a step; likewise, the decay time (*t_decay_*) is defined as the time interval for the current to fall from 90% to 10% of its final value [30]. Regarding the transient response, in the case of when one process is only involved in the generation and recombination of electron-hole pairs, a single time constant function can be used to fit the step curve. Therefore, we fitted the experimental transient curves by using a growth and decay exponential function with a single time constant [31]. Therefore, the rise and decay time constants were estimated using the following Equations (3) and (4), respectively [32].

(3) $$I_{photo}=I_{dark}+A\left[e^{\left(\frac{t}{\tau_{1}}\right)}\right],$$

(4) $$I_{photo}=I_{dark}+A\left[e^{\left(\frac{-t}{\tau_{2}}\right)}\right],$$

where *I_photo_* stands for the photocurrent and *I_dark_* the dark current, *A* is the proportionality constant, *τ_1_* and *τ_2_* are the rise and decay time constants, respectively, and *t* is the time at which the pulse starts. Figure 15 shows the time-resolved photoresponse to the white light irradiation.

After the fitting, it was found that the rise and decay time constants, corresponding to the Au/SRO/Si structure with the SRO A1 film, had the values of 114.89 and 146.72 ms, respectively. The smallest time constant (rise) is related to the generation and recombination time of hole-electron pairs, while the greatest one (fall) is related to the trap capture and release process time [31]. Table 5 lists the calculated rise and fall time constants for the A1 film Au/SRO/Si structure.

**Figure 16:**
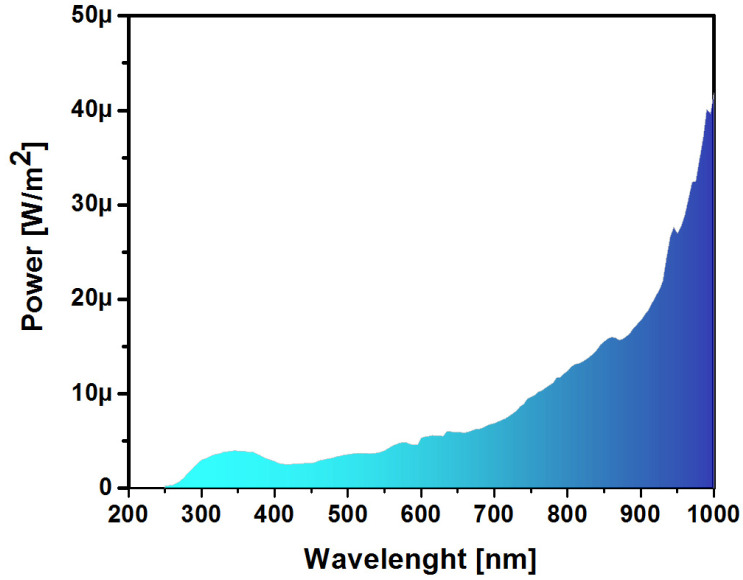
Light power pattern of the monochromator used as a function of the wavelength.

As can be seen in Table 5, the structure with thin film A1 exhibited the lowest rise and fall values. As the SSD of the SRO film increased, the time response became slower due to the fact that the thicker the films, the greater the concentration of hole-electron carriers involved in the generation-recombination and the trap capture and release processes. On the other hand, the responsivity parameter is a very important one in devices intended to be used as photodetectors [33]. This parameter allows understanding the electrical responses of the structure to different wavelengths of the incident radiation. To obtain this parameter, it is necessary to perform an I-λ measurement in the reverse polarized structure at −4 V by scanning light of different wavelengths in the range from 250 to 1000 nm. These measurements were made following the power pattern shown in Figure 2, where the used power of the incident light was obtained using a KILT-0950 spectroradiometer (International Light Technologies, Peabody, MA, USA). Figure 16 shows a graph of the optical power measured by the monochromator.

**Figure 17:**
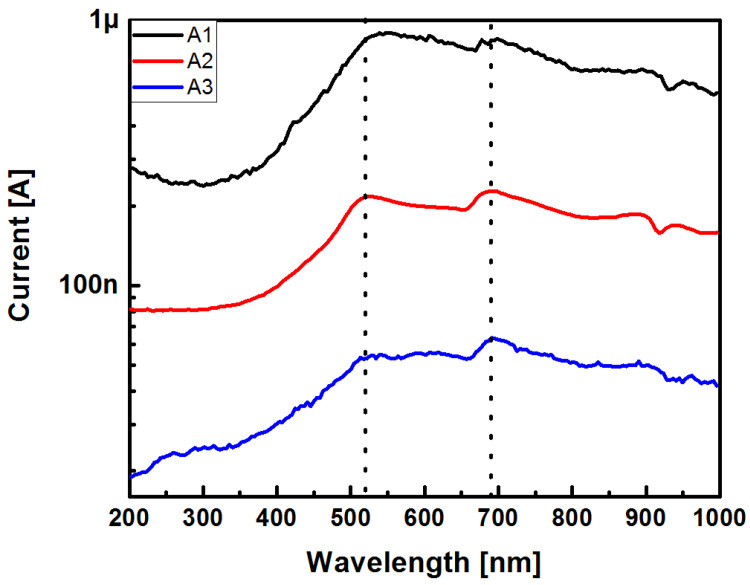
Curves I-λ of the Au/SRO/Si structures when reverse biased at −4 V as a function of the incident light wavelength.

Figure 17 shows the current responses of the SRO A1, A2, and A3 film structures as a function of the incident light wavelength under reverse bias at −4 V.

Figure 17 presents the I-λ curves of the Au/SRO/Si structures; these curves were similar in their structure but different in quantity. All curves were peaked at 520 nm and 690 nm in the spectrum region where they reached their highest intensity. There was a peak near 900 nm; that peak was due to photo effects in NPs:SiO_2_ films acting as traps [34]. It was also observed that when increasing the SSD parameter of the SRO film, the current was reduced, meaning that the optical response also decreased; however, as shown in Figure 17, the optical power obtained from the monochromator was not the same for the wavelength range measured. This fact forced us to look for a better method to investigate the photoresponse of the Au/SRO/Si structures to light. The method was through calculating the responsivity of the structures; the latter is one of the most fundamental properties of any detector, which is defined as the output signal per unit input, and it is obtained by using the photocurrent (*I_ph_*) and the incident optical power (*P_opt_*) at a specific wavelength λ whose relationship is given by Equation (5).

(5) $$\mathrm{Responsivity}=\frac{I_{ph}}{P_{opt}},\;\;$$

**Figure 18:**
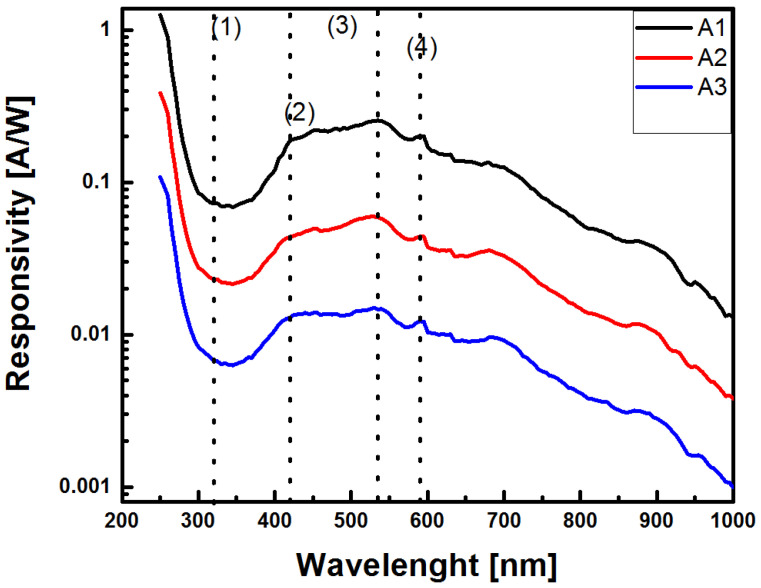
Responsivity of Au/SRO/Si structures at −4 V.

Figure 18 shows the responsivity behavior obtained for the Au/SRO/Si structures at −4 V. It was observed for the three structures that a high responsivity was kept below 300 nm (1), and it decreased, reaching a minimum in the vicinity of 300 nm. It again grew until it reached its maximum values in the range between 420 nm (2) and 535 nm (3) and then began to decrease, reaching a peak at 590 nm (4). The responsivity followed a similar behavior as the photoresponse shown in Figure 17; it decreased with increasing the SSD of the SRO film.

Select responsivity values are shown in Table 6, and it can be seen that for each structure, they were similar in the range between 420 nm and 590 nm, a fact that shows the highest photodetection area for the Au/SRO/Si structures.

**Figure 19:**
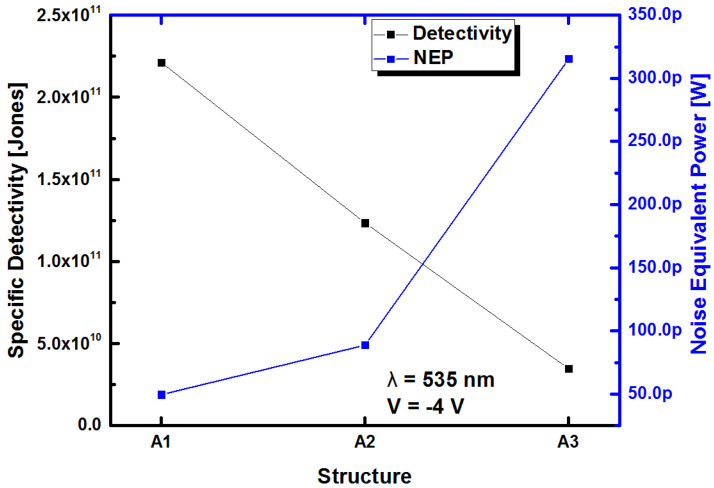
Specific Detectivity and noise equivalent characteristics of the Au/SRO/Si structures.

Specific detectivity (D*) is the key parameter for characterizing the photosensitive performance of photodetectors, which is a measure of the noise in the device. D* is obtained by Equation (6):

(6) $$D^{*}=\frac{R_{p}}{\sqrt{2eJ_{dark}}},\;\;\;$$

where *R_p_* is the peak responsivity, *e* is the electron charge magnitude, and *J_dark_* is the current density in the dark, including all possible noise in the device under the same measurement conditions. Given the mode of operation of the analyzed photodetector, where shot noise is the dominating source when the device is operating in photoconductive (biased) mode, the dark current is dominated by the shot noise; for that, we used the definition of D* shown in reference [35]. Although the responsivity effectively defines the sensitivity of a device, it gives no indication of the minimum radiant flux that can be detected. This minimum detectable flux is defined as a signal-to-noise ratio of unity. The latter defines the NEP (noise equivalent power), which is another important parameter for evaluating photodetector performance. NEP is defined as the minimum power that a detector can clearly detect. The noise equivalent power of any detector is obtained from Equation (7) [31,32]:

(7) $$NEP=\sqrt{\frac{A\left(F\right)}{D^{*}}}\;\;\;\;\;$$

where *A* is the area of the detector (0.12 cm^2^), and F is the noise band wide of the detector, defined by Equation (8) [36]:

(8) $$F=\frac{0.35}{t_{r}},\;\;$$

where *t_r_* is the response time of the detector. From these relationships, D* and NEP can be calculated at an operating voltage of −4 V and a wavelength of 535 nm, which is the maximum peak of responsivity, as shown in Figure 18. The results of these parameters are shown in Figure 19.

Figure 19 shows the D* variation as a function of the different Au/SRO/Si structures, where the structure with the best D* was observed to be A1, with $D^{*}=2.21\times 10^{11}$ cm Hz^1/2^ W^−1^, decreasing with increasing the SSD in the SRO films. On the other hand, a decrease in NEP indicates an improvement in performance, corresponding to the highest D*, and the lowest value of NEP was that of the A1 Au/SRO/Si structure. Table 7 shows the calculated D* and NEP values for the Au/SRO/Si structures.

It is observed from Table 7 that the lowest value of D* corresponds to the highest value of NEP and vice versa; this fact confirms that the A1 structure exhibited the best photodetection performance at 535 nm.

## 4. Discussion

The Au/SRO/Si structures exhibited photodetection under white light incident radiation, as shown in Figure 9 and Figure 12. To explain photosensitivity, several factors must be considered. The first contribution is the dispersion of incident photons in areas not covered by gold contacts, interaction with photogenerated electrons [37], and the result of the charge-discharge process of Si-ncs [26], where the absorption of light (photon density) generates electron-hole pairs as a consequence of the discharge process from the Si-ncs, and a photocurrent is generated in the structure. Second, due to the nature of the SRO material, it shows interesting electrical properties, such as charge trapping and non-trapping; due to the defects mentioned in photoluminescence, these allowable electronic states placed within the wide band gap of the SRO material facilitate the photodetection of light signals in MIS-type structures, and moreover, induce the formation of P-N intrinsic regions [6] when it is reversely polarized. This creates a larger depletion space charge region that can absorb incident photons to create electron-hole pairs, thereby increasing the photocurrent [38].

**Figure 20:**
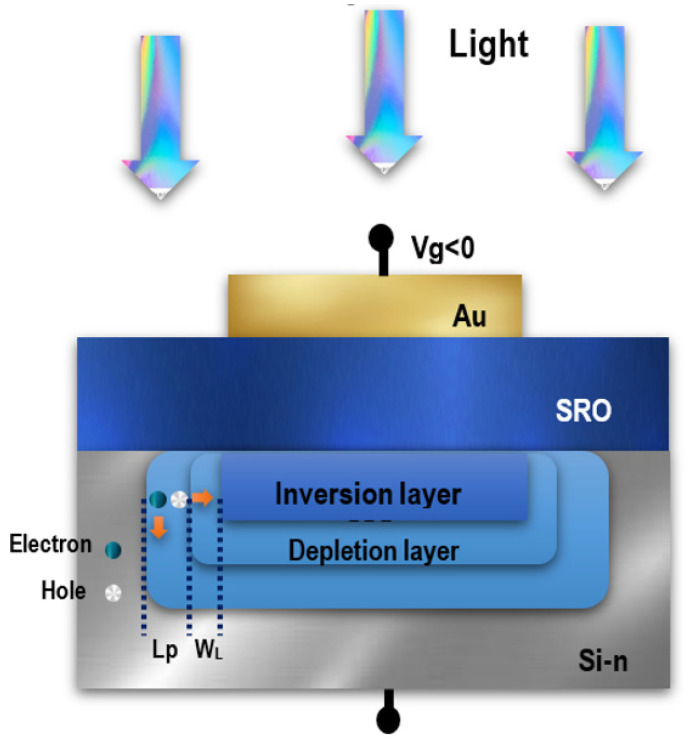
Schematic view of the photocarrier generation zones in the Si substrate and SRO layer.

There are three possible regions where incident light can generate photogenerated carriers: the lateral region of the depletion region (W_L_), the region around the depletion layer, where photogenerated holes can diffuse into the depletion layer (L_P_), and the SRO film around the Au contact [39], as shown in Figure 20. This can be explained by photogenerated carriers in the SRO thin film outside the gold contact area, which facilitate current flow through the oxide. Due to the applied electric field (negative voltage in the gold contact and positive voltage in the back contact), part of the photogenerated carriers diffuse under the contact. Likewise, these carriers are separated by an electric field and can tunnel through the Si-ncs, resulting in increased current flow through the MIS structure [16,37,38,39,40]. As mentioned above, the device responded well to light regardless of the opaque surface of the contacts, so another type of transparent contacts must be investigated, where the responsivity may be much greater than that shown in Figure 18; this is because gold contacts have high reflectivity in the visible infrared range (50% to 95% from 500 nm to 900 nm, respectively) [41], so at wavelengths greater than 500 nm, light may not penetrate into the contacts.

Furthermore, the fact that photoluminescence was observed in SRO suggests that the high-energy light can assist the movement of electrons, as UV light is able to pass through the Au contacts; this light is absorbed by the SRO, and it can contribute to a higher structure photocurrent. In addition, silicon is known to have a higher absorption coefficient at short wavelengths than at long wavelengths. Therefore, SRO samples had photoluminescence at short wavelengths between 500 nm and 850 nm. Photons with this wavelength could be absorbed by the induced P-N junction formed at the interface between the SRO film and n-type silicon, as shown in Figure 20. This property suggests that the Au/SRO/Si structure may be better suited to generate the “down-conversion” effect, which then contributes to the absorption of these photodetector devices [6]. The emission mechanism present in the PL spectra is related to some defects, such as neutral oxygen vacancies (NOV), weak oxygen bonds (WOB), non-bonded hollow oxygen centers (NBOHC), and positively charged oxygen vacancies [42,43,44,45,46,47,48]. These defects are the main centers of radiative recombination, and these ones contribute to the absorption of photons and the generation of electron-hole pairs, improving the photodetection of the Au/SRO/Si structure [18].

**Figure 21:**
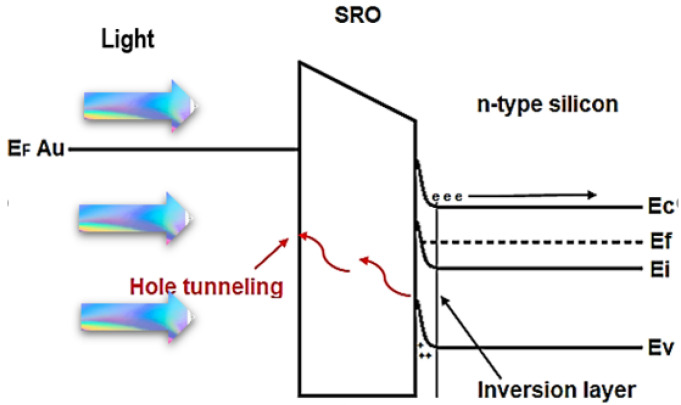
Schematic energy band diagram of Au/SRO/n-Si device under illumination.

On the other hand, when the band gaps of the unannealed SRO films were correlated, as shown in Table 3, whose values ranged from 2.2 eV to 2.5 eV, the maximum value was obtained in the responsivity, and it can be seen that the main contribution to the response was due to the interband transitions in the Si-ncs immersed in the SRO film with a band gap energy of 2.2 eV [49], which is consistent with the band gap of the film A2. From the photoluminescence measurements shown in Figure 7, it is possible to summarize the defects present in the films, which were involved in the absorption of light through the defects present in the SRO films (WOB and NBOHC) and contributed to photodetection [50]. The responsivity spectrum shown in Figure 18 confirms that, since the photoluminescence defect corresponded to the region of maximum responsivity of the Au/SRO/Si structure. The existence of defects as recombination centers in the SRO films may be the main reason for obtaining the response time values for the Au/SRO/Si structure, as shown in Table 5, which can be interpreted as the stoichiometric films of SRO. The peak corresponding to the vibrational mode S provides information about the composition of the deposited film. When this peak shifted towards higher wavenumbers, it indicated that the density of Si-O-Si bonds increases and that such films are more stoichiometric [1]. In the thin films, the peaks of the tensile vibrational modes were shifted to smaller wavenumbers, indicating a higher proportion of silicon in the amorphous phase. In this work, Si-O-Si bonds increased with increasing the dfs, where A1 was the least stoichiometrically thin film, and A3 was the most [1]. These Si-O-Si bonds provide shunting paths that reduce the photocarrier collection rate. Thus, higher charge separation efficiency in larger silicon clusters results in a fast response to exposure [51]. For structures A1, A2, and A3, the conduction mechanism of the injected current corresponded to the hopping mechanism. This assumption is based on the fact that the SSD deposition parameters are related to the film structure, where for films deposited with shorter SSDs, there are more defects, which are given by the oxidation states present [1], and on the fact that it had a smaller amount of hydrogen to passivate these defects. Thus, charge transport was dominated by assisted tunneling events (“hops”) in a large number of local defect states that acted as quantum wells separated by very small spatial regions, allowing tunneling between them, as we can see in Figure 21.

On the other hand, D* exhibited the highest value at the wavelength of 535 nm, as shown in Table 7, and the Au/SRO/Si structure and A1 film exhibited the highest value at the wavelength of 535 nm, as reported by Zacharias et al. [52]; this is due to the presence of silicon clusters. For this reason, we confirm that the SRO film exhibited these clusters due to the growth temperature of the film. Another aspect to highlight is that D* decreased with increasing the SSD, which may be related to the increasing thickness of SRO foil, as shown in Table 2. In addition, D* was mainly due to the low dark current of the Au/SRO/Si under reverse bias. However, the dark current was ultimately limited by the recombination current, which is an inherent property of SRO films, due to the presence of recombination centers, identified by PL spectroscopy. Furthermore, the two wavelengths, 590 nm and 420 nm, were due to Si-ncs with a diameter of 3 nm at 590 nm [53], while for 420 nm, it was due to excess silicon defects [54].

## 5. Conclusions

The photodetection effect was reported on Au/SRO/Si-n MIS structures deposited with SRO as an insulator and Au film as metal, with better results for structures with SRO films unannealed and films deposited at a shorter SSD. With the unannealed A2 structure, characterized by the 7 mm SSD and 255 nm thickness, the highest photocurrent response was obtained, where the white light photogenerated current increased by three orders of magnitude relative to the current in the dark, as confirmed by the I-t curve. The responsivity of the structures was obtained; they had similar shapes, with the best response in the region of 420 to 590 nm. The switching response of the structure showed a rise time of 120 ms and a decay time of 150 ms; as the SSD of the SRO film increased, the time response became slower. The D* and NEP values of the structures were obtained, and the structure with the SRO A1 film showed the best results at 535 nm. To better understand the presented phenomenon, various characterizations of the SRO films were performed, showing the differences between the studied films, where the atomic reorganization of the material occurred due to the annealing effect, and the shorter SSD was related to an increase in responsivity. Therefore, the Au/SRO/Si structure without annealing can be used as a photodetector, covering a wide spectral range. Hence, these structures are promising to be used as photodetectors in the visible light range with high sensitivity, high responsivity, and a low response time.

## Figures and Tables

**Table 1:** Description of the six SRO film labels.

50 sccm Hydrogen Flow Level		
Source-Substrate Distance	Without Annealing	With Annealing
6 mm	A1	A1’
7 mm	A2	A2’
8 mm	A3	A3’

**Table 2:** Thicknesses of the SRO films.

Sample	Thickness [nm]
A1	247
A2	255
A3	385
A1’	205
A2’	226
A3’	381

**Table 3:** Energy values of the band gap of SRO films without and with annealing.

Sample	Without Annealing	With Annealing
A1	2.5 eV	2.9 eV
A2	2.2 eV	2.7 eV
A3	2.4 eV	2.8 eV

**Table 4:** PL deconvoluted bands and their proposed source of emission.

	WOB ≈ 415 nm	NOV ≈ 460 nm	$E_{\delta }^{\prime }\;\approx \;520\;\mathrm{nm}$	NBOHC ≈ 630 nm	QC ≈ 760 nm	CLI ≈ 845 nm
A1	421		565	664		
A2	412-		531			
A3	411	472		610		
A1’	420				775	820
A2’	413		495		750	
A3’	417				791	

**Table 5:** Time constants of the A1 film Au/SRO/Si structure.

	*t_rise_*	*t_decay_*
A1	114.89 ms	146.72 ms
A2	120.73 ms	150.73 ms
A3	124.4 ms	166.57 ms

**Table 6:** Responsivity of Au/SRO/Si structures at different wavelengths.

	250 nm	420 nm	535 nm	590 nm
A1	1.25 A/W	188 mA/W	254 mA/W	200 mA/W
A2	386 mA/W	44 mA/W	59 mA/W	44 mA/W
A3	108 mA/W	13 mA/W	14 mA/W	12 mA/W

**Table 7:** Detectivity (D*) and noise equivalent power (NEP) of the Au/SRO/Si structures at different wavelengths.

	D* [cm Hz^1/2^ W^−1^]			NEP [pW]		
	420 nm	535 nm	590 nm	420 nm	535 nm	590 nm
A1	1.63 × 10^11^	2.21 × 10^11^	1.74 × 10^11^	69	49	62
A2	9.14 × 10^10^	1.23 × 10^11^	9.21 × 10^10^	119	88	110
A3	3.07 × 10^10^	3.47 × 10^10^	2.89 × 10^10^	350	310	378
